# Beyond the agenda: meetings as foundational to team entitativity, innovation, and effectiveness in scientific contexts

**DOI:** 10.3389/fpsyg.2026.1761061

**Published:** 2026-10-07

**Authors:** Deborah DiazGranados, Joseph A. Allen, Anita Blanchard

**Affiliations:** 1 Department of Psychiatry, School of Medicine, Virginia Commonwealth University, Richmond, VA, United States; 2 Division of Occupational and Environmental Health, University of Utah Health, Salt Lake City, UT, United States; 3 Department of Psychological Science, University of North Carolina, Charlotte, NC, United States

**Keywords:** entitativity, knowledge integration, meeting science, science of team science (SciTS), team decay, Team Development Interventions (TDIs)

## Abstract

Effective scientific collaboration is essential for addressing society’s most dynamic and interconnected problems, yet the interpersonal and structural processes that foster such collaboration are often undermined by fragmentation and knowledge heterogeneity. While the science of team science has identified the structural barriers to collaboration, less attention has been paid to the primary mechanism where these barriers are negotiated: the team meeting. Although frequently dismissed as an administrative tax on productivity, we argue that the meeting must be re-conceptualized as the essential structuring episode where a collection of experts transforms into a coherent entity. Drawing on structuration theory and the concept of entitativity–the perception of a social unit as a unified group–we propose a conceptual model positioning the meeting as the central arena for identity construction and knowledge integration. We then translate the model into actionable guidance for science meetings. Furthermore, we challenge the view of team development as an episodic activity, arguing instead that meetings function as continuous, embedded Team Development Interventions (TDIs). By enforcing a temporal rhythm of interaction, well-designed meetings serve as the critical counter-measure to team decay, preventing the erosion of shared mental models and trust that occurs when members retreat to disciplinary silos. We integrate insights from meeting science, team dynamics, and knowledge integration research to translate this theory into practical strategies. We conclude by offering future research directions to empirically study the mechanisms that can ultimately demonstrate how strategic meeting design can unlock the full integrative capacity of scientific teams.

## Introduction

Effective scientific collaboration is essential for addressing society’s most complex problems, yet the interpersonal and structural processes that foster such collaboration remain incompletely understood. Large-scale, multidisciplinary projects, whether in biomedical discovery, engineering innovation, or organizational/management science, require individuals from diverse disciplines to coordinate effort, share expertise, and integrate knowledge over time (Lotrecchiano et al., 2021; Stokols et al., 2008; National Academies of Sciences, Engineering, and Medicine [NASEM], 2025). While years of meaningful research in the science of team science (SciTS) have clarified how diversity of knowledge, leadership, and collaboration structures shape performance, comparatively little attention has been paid to the underlying psychological processes that transform a collection of scientists into a coherent and unified team. One such process, team entitativity, or the perception of a social unit as a cohesive, bounded entity (Campbell, 1958), is foundational to effective team collaboration, but remains under-theorized in scientific team contexts (Crawford and Salaman, 2012; Vangrieken et al., 2013). Because of recent advances in measurement (Blanchard et al., 2020), the tools to assess entitativity are providing preliminary evidence of its importance through exploration of team meetings (Blanchard et al., 2022).

Despite being the most frequent and structured forum for interaction in scientific teams, meetings are often viewed as logistical necessities, rather than as the crucibles in which collective identity is constructed and maintained (Allen et al., 2015). Meeting science research has challenged this narrow view, positioning meetings as central, dynamic contexts for meaning-making, relationship building, and coordination (Allen and Lehmann-Willenbrock, 2023; Allen and Rogelberg, 2013). Recently, Scott and Allen (2023) advanced the idea that meetings are microcosms of organizational life, reflecting and shaping broader cultural, structural, and identity processes. Within scientific teams, meetings provide recurring opportunities to establish boundaries, reinforce shared goals, and revisit the team’s evolving history. Interestingly, these are consistent with the three features Blanchard et al. (2020) identify as critical antecedents of entitativity. Consequently, meetings may represent not only coordination mechanisms but identity-building events that shape how members understand themselves in relation to one another and the team’s overarching mission.

To move the literature forward, there is a need to conceptually explore the underlying principles that drive effective meetings, and to examine how these principles align with existing formal theories, assessing which theories are best suited to this context, and where existing theories may need to evolve to advance the field. Marks et al.’s (2001) temporal framework of team processes offers a useful scaffold, that meetings span both transition phases (e.g., goal setting, planning, strategy formulation) and action phases (e.g., coordination, performance monitoring, reflection). We believe that meetings do not inherently possess the features of effective Team Development Interventions (TDIs)–mechanisms that improve team functioning through structured interaction, feedback, and learning (Shuffler et al., 2018). Left unstructured, meetings can lean toward a different set of tendencies like reproducing existing status hierarchies, defaulting to passive information sharing rather than integration, and interruption of focused work (Rogelberg et al., 2006). These outcomes require no deliberate effort to emerge, they are simply what results when colleagues gather without structure. However, when intentionally designed, meetings can serve as ongoing, embedded TDIs that strengthen interdependence, clarify roles, promote trust, and reinforce shared mental models. This perspective reframes meetings not as interruptions to real work but as vital parts of the work itself through which teams adapt, learn, and evolve. Given the wide variety of meeting types and structures across scientific teams, from small project teams to advisory teams, to multisite, multi-level centers (Hall et al., 2012), we define some boundary conditions of our recommendations in the section on the science of meetings.

The existing literature on SciTS, team processes, and meeting science remain largely disconnected regarding how routine, high-quality meetings contribute to the emergence of team entitativity and, ultimately, to innovation and effectiveness. Theoretical integration is needed to explain how interaction patterns within meetings, particularly those that emphasize shared goals, mutual accountability, and reflective communication, can shape team identity over time. Drawing upon theories of group processes (Salas et al., 2005), structuration theory, and empirical findings from both team and meeting science, the purpose of this paper is to both propose a conceptual model that positions meetings as foundational mechanisms for developing team entitativity in scientific team contexts and provide actionable guidance for science teams to implement during their meetings. Specifically, we argue that repeated, intentional meeting interactions create a recursive cycle in which shared understanding, interdependence, and collective purpose co-evolve to strengthen perceived unity and enable greater innovation and effectiveness. In doing so, this manuscript bridges meeting science, entitativity theory, and team development research to provide a novel framework for understanding how scientific teams can strategically leverage their meetings as ongoing interventions for cultivating strong, cohesive, and high-performing collaborations.

## The science of meetings

Meetings are arguably the most ubiquitous, yet often maligned, feature of organizational life (Rogelberg et al., 2007). The sheer magnitude of this activity underscores its importance and cost, with annual financial losses from unproductive meetings estimated at billions of dollars (Mroz et al., 2018). The knowledge worker bears the brunt of this investment and inefficiency. Recent data confirms this massive time commitment: the majority of U.S. workers spend three or more hours per week in meetings, with 59% of employees in large enterprise organizations dedicating five or more hours every day to scheduled gatherings (Calendly, 2024).

This time is further degraded by a constant state of fragmentation due to the interrupting nature of meetings (Rogelberg et al., 2006). Data from Microsoft (2025) show that during core work hours, the average employee is interrupted by meetings, emails, or pings every 2 min, adding up to an average of 275 interruptions a day. Continuing with this data, a significant contributor to this chaos is that 60% of all meetings are *ad hoc* rather than scheduled. A profound work-related capacity gap has emerged in that 80% of the global workforce, employees and leaders alike, now report lacking enough time or energy to complete their work, and nearly half of employees (48%) describe their work as feeling chaotic and fragmented. This centrality of meetings stands in stark contrast to employee dissatisfaction, as 45% of professionals now feel meetings actively reduce their overall productivity (Calendly, 2024). This significant and costly performance-satisfaction gap necessitates a systematic, scientific approach to understanding the underlying dynamics of the meeting phenomenon (Allen and Lehmann-Willenbrock, 2023) and developing evidence-based practices that improve team functioning.

The science of meetings is defined as the systematic study of what takes place just prior to, during, and right after a meeting (Allen et al., 2015). Its core proposition is that meeting quality is a manipulable variable that directly influences crucial individual, team, and organizational outcomes (Allen and Lehmann-Willenbrock, 2023). The science of meetings has emerged as a distinct, interdisciplinary field dedicated to rigorously studying meetings and the activities that take place in and around meetings, rather than treating them merely as a context for other research (Allen and Lehmann-Willenbrock, 2023). Drawing primarily from organizational psychology, communication studies, and management science, the field provides an empirical foundation for transforming meetings from a necessary evil into a critical productivity tool (Mroz et al., 2018). Previous research confirms that meetings shape both employee job satisfaction and engagement (Rogelberg et al., 2010; Allen and Rogelberg, 2013).

The relevance of meeting science becomes especially apparent in scientific team settings, where meetings are not simply routine coordination events but central sites of knowledge integration work. Scientific teams operate in complex problem spaces characterized by ambiguity, uncertainty, interdependence, long time horizons, and evolving goals (Hall et al., 2012). The problems these teams address are often ill-structured rather than routine. They require the integration of partial, specialized, and sometimes competing forms of expertise. Moreover, the membership of scientific teams is often diverse in ways that heighten both the value and difficulty of collaboration. Project teams may include principal investigators, co-investigators, methodologists, statisticians, clinicians, community partners, graduate students, postdoctoral scholars, research staff, and external stakeholders, each bringing different disciplinary assumptions, technical languages, epistemological commitments, institutional constraints, and status positions (Tebes and Thai, 2018). These conditions create precisely the kind of collaborative environment in which meeting quality matters most. When meetings are poorly designed, expertise remains siloed, assumptions remain implicit, and coordination problems accumulate. When meetings are well designed, they become mechanisms for shared sensemaking, knowledge integration, decision quality, team learning, and scientific innovation.

Rogelberg et al. (2025) note that meeting science explicitly differentiates itself from the broader teams and group research literature, which has historically overlooked meetings as an important phenomenon in its own right. Rather than viewing meetings as merely functional task episodes, meeting science recognizes them as social and symbolic spaces where “values, identities, and power are actively negotiated, shaped, and reified” (p. 16). This perspective is particularly important for science teams because meetings are often the place where disciplinary legitimacy, methodological preferences, authorship expectations, leadership influence, and the meaning of scientific contribution are negotiated. While the science of teams has informed key inputs and processes for creating effective teams, the meeting itself has often been overlooked as a key element for launching, sustaining, and optimizing scientific teamwork.

### Literature review

Recent reviews of meeting science literature highlight both the need to examine meetings across multiple levels of analysis (i.e., individual, team, and organizational; Allen and Lehmann-Willenbrock, 2023; Rogelberg et al., 2025), and the field’s lingering tendency to focus more heavily on individual experiences than on the complex contextual environments in which meetings occur. Meeting scholars have investigated contextual features to some extent, including interaction patterns and team dynamics (e.g., Kauffeld and Lehmann-Willenbrock, 2012), affective and behavioral sequences during meetings (Lehmann-Willenbrock et al., 2017), and structural meeting characteristics that shape employee attitudes (Lehmann-Willenbrock et al., 2016). However, these contributions only begin to address the broader, multi-layered contexts in which contemporary scientific teams operate.

A particular limitation is that much of the foundational meeting science literature has historically emphasized relatively generic meeting tasks (Allen et al., 2015). These include information sharing, status reporting, agenda review, routine coordination, planning and scheduling, problem identification, problem solving, brainstorming, decision making, feedback exchange, and follow-up assignment. These task categories are essential and remain relevant to science teams. However, scientific collaboration also involves more specific and complex forms of meeting work. In team science, meetings may be used to develop theoretical models, reconcile disciplinary assumptions, translate concepts across fields, establish shared definitions, align methodological approaches, interpret ambiguous findings, integrate quantitative and qualitative evidence, adjudicate competing explanations, coordinate data collection protocols, manage authorship and intellectual contribution, address ethical or regulatory issues, and make consequential decisions about scientific direction. Thus, applying meeting science to team science requires moving beyond generic task classifications toward a more precise understanding of the scientific work being accomplished through meetings.

This distinction matters because meetings in scientific teams vary substantially by team type, project scope, and organizational level. In a project team of investigators, meetings are often focused on the direct conduct of the science (i.e., developing research questions, coordinating methods, solving analytic problems, interpreting results, preparing manuscripts and grants, and making decisions about the next phase of the project). These meetings tend to involve a relatively bounded set of investigators and staff who share responsibility for a specific scientific product or program of inquiry. In contrast, meetings in multisite studies or complex research centers often occur across multiple levels. A large center may include executive leadership meetings, steering committee meetings, site principal investigator meetings, core-specific meetings, workgroup meetings, community advisory meetings, data coordinating center meetings, and implementation or dissemination meetings. In these settings, meetings serve not only scientific integration but also governance, cross-site standardization, resource allocation, compliance, stakeholder management, and strategic alignment. The same meeting science principles may apply across these settings, but the mechanisms, constraints, and outcomes may differ substantially.

### Theoretical perspective

For this reason, the theoretical and practical claims in this paper should be interpreted within specific boundary conditions. The primary focus is the project team of investigators rather than the full ecology of meetings embedded in multisite initiatives, large research centers, or complex consortia. The focal meetings are those in which investigators and core project members engage directly in scientific collaboration, including knowledge sharing, interpretation, integration, coordination, and decision making. The assumptions and recommendations offered here are most applicable to knowledge-intensive scientific teams whose work requires interdependence among members, integration of diverse expertise, and recurring interaction over time. They are less directly applicable to purely administrative meetings, large informational briefings, ceremonial meetings, one-way dissemination meetings, or center-level governance meetings in which the primary task is oversight rather than direct scientific knowledge integration. They may also require adaptation for highly hierarchical teams, large multisite networks, virtual-only collaborations, community-engaged partnerships, and teams with fluid or rotating membership.

The lack of emphasis on complex team processes presents two major, interrelated limitations for applying meeting science to modern, knowledge-intensive scientific work. First, the field still suffers from conceptual fragmentation, lacking a unifying theory capable of predicting meeting outcomes across the highly complex and varying contexts found in research and development, which limits its utility for designing interventions specific to knowledge work (Allen and Lehmann-Willenbrock, 2023). Second, the historic focus on generic tasks means the existing literature has not adequately addressed the unique challenges of scientific collaboration, which often involves fluid team membership, unevenly distributed expertise, and the requirement for deep, nuanced sharing of highly specialized information (Rogelberg et al., 2025).

These gaps become especially visible when considering how science teams operate at the intersection of diverse expertise. For many scientific teams, knowledge integration–the process of synthesizing non-overlapping information from different domains to generate novel insights–is not a peripheral activity but the primary collaborative task. In this context, meetings are not merely for status updates or routine coordination. They are the place where individual expert knowledge must be shared, questioned, translated, critically evaluated, and integrated into new concepts, methods, interpretations, and products. Therefore, the core conceptual gap lies in understanding how meeting design, meeting leadership, and emergent interaction structures facilitate or inhibit this complex integration process.

Current inquiry must shift more directly to the internal dynamics of the meeting as a self-organizing knowledge system. This includes the need to move beyond traditional metrics of meeting participation, which may fail to accurately assess engagement in scientific teams whose size, composition, expertise distribution, and power dynamics can change over time. For example, equal speaking time may not always be the optimal indicator of meeting effectiveness when expertise is highly specialized and unevenly distributed. At the same time, persistent participation imbalance may signal that certain forms of expertise, methodological concern, or stakeholder knowledge are being marginalized. New methods are required to reliably link participation patterns, expertise utilization, psychological safety (Edmondson and Lei, 2014), and team outcomes, particularly in environments where expert contribution is highly valuable but not always evenly distributed (Rogelberg et al., 2007).

The effectiveness of a meeting for science teams hinges on the capacity of the team to pool, translate, challenge, and integrate highly specialized information. This highlights the critical need for research that explicitly links meeting leadership, interaction behavior, structural design, and contextual conditions to the development of meetings as places where scientific teams can share freely, understand colleagues’ perspectives and approaches, and build on one another’s expertise. Under these conditions, meetings become more than a coordination mechanism. They become central infrastructure for scientific collaboration, collective learning, and innovation.

## Meetings are a venue of scientific collaboration

To understand why meetings are a critical mechanism for team development, we must first establish the unique process demands that exist in scientific collaboration. Unlike teams engaged in routine or production-oriented tasks, science teams are defined by their effort to address scientific challenges, leveraging varied expertise and perspectives from members of the team. The ultimate, complex goal is knowledge integration. Hall et al. (2012) describe the purpose of transdisciplinary research as the need to “integrate and ultimately extend beyond discipline-specific concepts, approaches, and methods to accelerate innovations” (p. 415). This act of integration is not a simple exchange of information; it requires engaging in complex social and cognitive processes where team members have to discuss and present deep-seated disciplinary assumptions, which may need to be negotiated and synthesized into a new and shared conceptual framework.

Meetings, then, are the primary, recurring context where this social foundation can be built, tested, and maintained. The science of meetings states that these gatherings are far more than just instrumental tasks; they are essential for building community (Tracy and Dimock, 2004). The time spent in meetings serves as a space for sharing one’s own professional identity and where collective identities can be created. This perspective reframes the meeting as the central, structured social arena for the scientific team. The socio-emotional foundation required by science teams cannot be built over email or in the abstract. It is created (or destroyed) in the real-time, high-stakes interactions of a meeting.

This social foundation, built through relational meeting behaviors, directly enables the complex cognitive work essential for scientific innovation. Meetings are then viewed as the primary space where effective communication should occur (Rogelberg et al., 2007) and where true collaboration (Allen and Lehmann-Willenbrock, 2023) and knowledge integration (Hall et al., 2012; Salazar et al., 2012) must occur. The science of meetings provides a specific vocabulary for these functions. For example, Allen and Lehmann-Willenbrock (2023) identify the relating features of meetings (e.g., managing conflict, building relationships, fostering inclusivity) as a core feature that directly builds the socio-emotional foundation. This foundation, in turn, is the platform for interacting (e.g., sharing information, challenging ideas) and engaging (e.g., member motivation and participation). This perspective is crucial because the negative emotions often associated with meetings (Mroz et al., 2018; Microsoft, 2025) are particularly detrimental to the high-stakes work of science teams.

To harness the potential of scientific teams, we should confront the pervasive but paradoxical nature of meetings. In the context of science, meetings are frequently viewed as a tax on productivity, time spent away from the real work of writing, experimentation, or analysis (Mroz et al., 2018). However, this view is fundamentally flawed. As Godemann (2008) argues, the core challenge of collaborating across disciplines is not the generation of data, but the integration of highly distributed and heterogeneous knowledge. Zhang (2023) expands on this, highlighting that science teams are defined by their knowledge heterogeneity, a state where expertise is highly distributed and distinct among members. While this heterogeneity is critical for solving complex problems, it cannot be leveraged in isolation; it requires social mechanisms to bridge the gaps between non-overlapping domains. Therefore, the meeting must be re-conceptualized: it is not a distraction from the science; it is the primary temporal and physical space where diverse knowledge is actively synthesized. It is the essential venue where the good of scientific collaboration, the fusion of diverse expertise, must overcome the bad of logistical fragmentation.

This re-conceptualization requires viewing the meeting as the primary mechanism for creating cognitive salience within a necessary temporal workflow. In the dispersed nature of modern science, team members are often deeply entrenched in their specific disciplinary thinking (Hall et al., 2012), focused on their traditional theories, frameworks, and methodologies. The meeting functions as a critical temporal punctuation, giving the team the space to focus on attending to the same problem, at the same time, with the same level of focus. This structure is essential because science teams must successfully navigate a rhythm of distinct team processes (Marks et al., 2001). Meetings provide the necessary space to engage in transition phases (i.e., developing shared vision, setting goals) as well as action phases (i.e., coordinating tasks, monitoring progress). For science teams, the meeting-based transition phases are particularly critical. Pennington (2016) suggests that successful integration requires participatory processes where team members engage in the active, real-time negotiation of meaning to identify and resolve representational gaps. Without the enforced salience of the meeting, team members remain isolated in their individual learning loops; the meeting is the structural bridge that converts individual learning into group-level knowledge (Pennington, 2016; Salazar et al., 2012).

## How meetings serve a strategic pivot point?

Meetings themselves have recently emerged as an important proximal driver of entitativity. Empirical work shows that meeting features–such as equitable participation, high-quality leader facilitation, and strong relevance of agenda items to members’ work–contribute to stronger perceptions of the group as a coherent unit (Blanchard et al., 2022). When meetings function effectively as structuring episodes, they not only reinforce perceptions of group unity but also yield downstream benefits such as enhanced psychological voice, higher evaluations of meeting effectiveness, and greater meeting satisfaction. In this way, meetings serve a dual purpose, they are both the mechanism through which the group’s social structure is enacted and the context in which members come to perceive that structure as real, stable, and meaningful.

Given that the meeting is the primary context where the team’s foundation is built and where cognitive synthesis occurs, the effectiveness of these gatherings becomes a critical variable in scientific success. It is insufficient to merely gather experts in a room and hope for integration; the benign neglect of meeting design that characterizes many organizations (Rogelberg et al., 2007) is particularly perilous for science teams. If the meeting is the venue where values, identities, and power are actively negotiated (Rogelberg et al., 2025), then a poorly managed meeting is not just a waste of time, it is a missed opportunity to innovate and integrate knowledge required for scientific progress.

Therefore, science teams must leverage the insights from meeting science to intentionally design these interactions. Ben-Menahem et al. (2016) highlight that knowledge creation requires a specific rhythm of coordination, moving between differentiation (i.e., individual, specialized work) and integration (i.e., collective synthesis). An effective meeting serves as the structural pivot point in this rhythm. It is the specific event where the team moves from independent thinking to collective sensemaking. By applying meeting science principles, such as facilitating for inclusion, managing time for deep discussion, and structuring conflict, leaders can ensure that this pivot is successful. In doing so, the meeting creates the dynamic space where knowledge is not just exchanged, but transformed, enabling the team to achieve the deep integration that transdisciplinary science demands (Salazar and Lant, 2018; Hall et al., 2012).

## The structuration process of team entitativity through meetings

Structuration theory (Giddens, 1984) provides a particularly powerful metatheoretical lens for understanding how meetings function as the crucible in which groups are formed, maintained, and transformed. At its core, structuration theory emphasizes the duality of structure–that is, the idea that social structures both shape and are shaped by the ongoing actions and interactions of individuals. Agency and structure are mutually constructing, individuals’ behavioral choices create and recreate the social arrangements (e.g., norms, roles, expectations) that subsequently guide and constrain future behavior. From this view, groups are not fixed, self-contained entities; rather, they are continuously produced and reproduced through members’ patterned interactions over time.

Meetings are the primary venue in which this recursive process of structuration unfolds within workgroups. They are discrete, time-bound episodes during which members come together to engage in collective sensemaking, negotiate rules and roles, and allocate resources–both material and symbolic. These moments of interaction effectively do the work of creating the group as a social structure and, in turn, reinforcing members’ identities as part of that group. Thus, meetings serve as recurrent structuring episodes that allow group members to enact, refine, and stabilize the very social entity they inhabit.

While structuration theory reminds us that groups are socially constructed rather than objectively permanent, recent advances in social cognition illuminate why the perceived solidity of the group matters so profoundly. The construct of entitativity (Campbell, 1958; Lickel et al., 2001), defined as the extent to which a collection of individuals is perceived as a coherent, unified group, provides an essential complement to structuration theory within the context of meeting science. Entitativity reflects the degree to which members cognitively reify the group–treating it as a real, stable, meaningful unit that exists above and beyond the individuals who compose it.

In this way, entitativity can be viewed as the degree of structure crystallization resulting from the ongoing enactment processes described by structuration theory. When interaction patterns, shared norms, and collective goals become sufficiently established through repeated structuring episodes (i.e., meetings), members increasingly experience the group as a cohesive entity. Antecedents of entitativity–such as interaction quality, perceived similarity, shared goals, and coordinated behavior (Blanchard et al., 2020; Campbell, 1958; Lickel et al., 2001) –map directly onto the agency–structure dynamics central to structuration. Moreover, contemporary measures of entitativity provide researchers with a quantitative means to assess the strength of group reification, which has been linked to meaningful outcomes including identification, satisfaction, commitment, and prosocial behavior (Blanchard et al., 2022).

Collectively, integrating structuration theory with entitativity advances meeting science by clarifying how and why meetings matter for group functioning. Meetings offer the behavioral substrate through which groups are continually constructed, while entitativity captures the psychological consolidation of that structure. As such, meetings are not merely sites for coordination–they are generative episodes that build and strengthen the social fabric of workgroups, shaping both group outcomes and members’ experiences within them.

## Strategic meeting design as a Team Development Intervention (TDI)

While the need for team development for science teams is clear, the mechanism for delivery has historically been misplaced. Team Development Interventions (TDIs) are viewed as actions that alter the performance trajectories of organizational teams; they are intentional actions that can improve or support teams that may be experiencing conflict; they may maintain and sustain teams that are adequately performing, or grow and maximize the capacities of teams ready to perform at higher levels (Shuffler et al., 2018). While these distinct interventions have value, they suffer from issues of transfer and decay. In the context of science teams, decay refers to the inevitable erosion of shared mental models and interpersonal trust (Mayer et al., 1995) that occurs when members retreat from an initial project meeting back to their offices. The clarity of purpose and social cohesion built often degrades when faced with the daily friction of mismatched terminology, the cognitive fragmentation of juggling being on multiple teams, and the competing priorities that come from one’s workload.

To counteract this decay, we must operationalize our re-conceptualization of the meeting across the team’s lifecycle. We propose a shift in perspective: both the initial ideation sessions that may be required of teams and the routine team meetings should be viewed as the primary, continuous TDI. As we have argued, the meeting is the central arena where the team’s social foundation is built, and knowledge is integrated. When deliberately designed, the meeting can therefore fulfill the definition of a TDI–an intentional action to maintain and sustain team performance (Shuffler et al., 2018). This intervention function changes based on the meeting type. Initial ideation meetings act as a space where a team chartering intervention may be required, where the problem space is defined and the initial clarity of purpose and roles are established. Routine meetings then serve as the mechanism of continuous reinforcement. They enforce the temporal rhythm necessary to refresh shared mental models and repair interpersonal trust before they fully erode. Thus, by treating the entire system of meetings as embedded TDIs, leaders can leverage the ubiquity of these gatherings to inject development directly into the workflow, ensuring that the team’s integrative capacity is not just established once, but rigorously maintained.

The power of the meeting as a TDI lies in its ability to deliver team development concurrent to the task work. However, simply gathering the team is not an intervention; the design of the interaction determines the outcome. Meeting science demonstrates that specific design characteristics serve as the active ingredients of this intervention, directly moderating its effectiveness. A meeting that lacks intentional design, characterized by vague agendas, poor time management, or off-topic digressions, can serve as a negative intervention, actively eroding trust and entitativity (Rogelberg et al., 2025).

To function as a positive TDI, scientific team leaders must treat meeting design as a strategic lever. This involves moving beyond administrative logistics to implement evidence-based features that drive team development:

- **Relevance and Structure:** The use of a relevant agenda, circulated in advance, does more than organize time; it primes cognitive attention and reinforces the shared purpose of the team (Mroz et al., 2018). This structural clarity reduces ambiguity, directly supporting the team’s shared mental models and it allows team members to help build cognitive connections from their own discipline specific knowledge to the shared team goal.
- **Procedural Justice and Voice:** Active facilitation that ensures participation equality is not just being kind; it is a mechanism for building psychological safety. By designing interactions where all voices are heard, especially in multidisciplinary teams where power dynamics can silence team members who are the single representative of a discipline, leaders reinforce the team’s inclusive identity (Allen and Lehmann-Willenbrock, 2023).
- **Constructive Conflict Management:** Finally, the meeting design must create a safe space for intellectual disagreement. In science teams, the goal is integration, not consensus. A meeting designed to encourage productive task conflict (Costa et al., 2015) allows the team to negotiate deep-seated disciplinary assumptions without destroying interpersonal trust (Dirks and de Jong, 2022).

When these design features are present, the meeting transforms from a logistical burden into a powerful, recurring intervention. It becomes the core work itself through which scientific teams construct identity, adapt, and evolve toward greater effectiveness.

Building on the theoretical foundations outlined above, we now offer practical recommendations for team science practitioners seeking to improve meeting practices. We then close with a discussion of future research directions to further integrate the literature on meetings and the literature on the science of team science.

## Practical recommendations for improving the science team meeting

The re-conceptualization of the meeting as an embedded TDI requires a corresponding shift in practice. For scientific team leaders, this means moving from passive scheduling meetings to active designing interventions when planning for a meeting, and considering the stage of the team. We offer the following practical recommendations, organized by the team lifecycle, to operationalize these insights. Table 1 provides a summary of the recommendations which follow. We provide explicit examples of how to enact these recommendations as well as how they may differ for virtual and hybrid meetings.

**TABLE 1:** Summary of recommendations with considerations for virtual and hybrid formats.

Recommendation	Enactment	Considerations for hybrid/virtual teams
1a. Build interpersonal relationships	Use Icebreakers and other knowledge building interactions.	Hybrid and virtual teams will have at least some members who do not know each other and cannot interact outside of the meetings. It is essential to create relationships between the distributed group members.
1b. Establish norms of engagement	Create charters or explicit discussions of participant expectations.	Additional norms of meeting participation for virtual and hybrid meetings can include using mute, camera, and the chat function during the meeting.
2. Focus on meetings as opportunities for active participation and problem solving	Use question based agendas that will encourage problem-solving.	Consider using breakout rooms in larger virtual meetings so that all participants have an opportunity to contribute. Groups report ideas back to the larger group for additional discussion.
3. Focus on synthesizing and integrating	Meeting facilitator(s) should call on different participants’ expertise and explicitly create a cohesive whole.	Hybrid meetings may be particularly challenging for the distributed members to receive adequate attention and engagement. Facilitator(s) should explicitly call on members who are not in the room for their input.
4. Create a digital location to prevent knowledge atrophy between meetings	There should be one digital location that all members use to store any information or artifacts for the team. Facilitators must ensure people use this site for all storage.	For online teams in particular, this digital location can serve as a proxy for the missing shared physical space of face-to-face teams. It reinforces entitativity by tying members’ mental models to shared digital space.

### Chartering the meeting strategy

Just as science teams establish protocols for data collection, they must establish protocols for their interaction. This work is best situated in the team’s transition phase, when members are not yet executing tasks but are instead reflecting on and planning how they will work together. We recommend dedicating the first project meeting not to the science, but to first establishing an understanding of the other group members as well as the expertise and perspectives contributing to the science being conducted. Cross-disciplinary science teams require effective interpersonal skills (e.g., sensitivity and emotional engagement) in order to spark creativity Murugavel and Reiter-Palmon (2023) and productive research outcomes (Cheruvelil et al., 2014; Love et al., 2021). If the team is composed of people who do not know each other, or do not know each other well, some level of interpersonal relationship needs to be created, first to increase the perceptions of similarity necessary for entitativity, but also to increase trust that the norms that develop in the structuration process will be acceptable and relevant for the group. This interpersonal foundation is a necessary precondition for the psychological contract the team will build together. It is important to note that individuals cannot commit to shared expectations for a group they do not yet trust or recognize as an entity.

Once the necessary level of interpersonal understanding has developed, then the members should focus on co-creating the rules of engagement to explicitly answer critical questions such as: How will we handle conflict? Who will organize the meeting agenda? How will we ensure everyone on the team (e.g., community members, sole disciplinary team members) are heard? What is the specific purpose of our standing meetings? Without explicit rules of engagement, science teams risk defaulting to conventional academic norms that are not designed to meaningfully include a range of collaborators (Tebes and Thai, 2018). This establishes the initial entitativity boundaries and creates the psychological contract for future interactions (Köhler et al., 2013). Furthermore, creating a shared set of rules of engagement that can be adjusted throughout the life of the team helps to clarify these interaction norms upfront and can help mitigate the negative effects of status differences, which have been shown to increase surface acting and reduce psychological safety in meetings (Shumski Thomas et al., 2018). Explicitly co-creating rules of engagement gives members who may not hold status levels equal to other team members or those who only represent their discipline a legitimate agreed upon basis for raising concerns or asserting expertise, rather than relying on informal norms that tend to emphasize existing power differences. As such, the psychological contract established through the development of the charter functions as a unifying mechanism for the team. While charters are established during the transition phase, they should not be treated as static. Teams may need to revisit and renegotiate their rules of engagement once they move into the action phase and begin executing the science and tasks they are responsible for. This is particularly important if new members join the team, conflict or tensions emerge, or the original norms prove unworkable in practice.

**RECOMMENDATION 1**: During the transition phase, dedicate the initial meeting to building interpersonal understanding and co-creating explicit rules of engagement that define interaction norms and mitigate status differences and revisit these norms during the action phase as needed.

### Priming for cognitive salience

Administrative agendas (i.e., agendas that consist of structured lists of topics for a meeting) often fail to engage the attendees of a meeting and, most importantly, they often fail to utilize the diverse expertise of the team. This is particularly critical during the team’s action phase, when meetings are the primary venue through which members coordinate and execute the interdependent science itself. Further, they may not help the team develop the necessary structure to crystallize the team as an entity. As a solution to change the use of administrative agendas, agendas should shift to question-based agendas. Instead of listing topics, list the specific questions, challenges, or deliverables the meeting needs to address (e.g., What are the barriers to submitting the grant proposal? Or How do we resolve the discrepancy between Data A and Model B?).

Circulating these questions 48 h in advance creates the cognitive salience required for knowledge integration, forcing members to leave their disciplinary silos and engage with the shared problem before they enter the room (Mroz et al., 2018). Because action-phase meetings recur throughout the life of a project, this practice offers a repeatable structure teams can apply to each standing meeting, rather than a one-time intervention established at the onset of a project. Furthermore, Rogelberg et al. (2025) note that this practice enhances inclusivity and preparation; by clarifying the specific input required, it allows team members–regardless of their discipline or status–to process the information and come prepared with meaningful insights, transforming the meeting from a space of passive information consumption into one of active, collective problem-solving. Moreover, the meeting becomes outcome-focused and oriented to answering questions.

**RECOMMENDATION 2**: During the action phase, replace static topic lists with question-based agendas to prime cognitive salience and shift the meeting focus from passive reporting to active, inclusive problem-solving.

### Facilitate for integration

For science teams working toward a shared outcome, whether drafting a manuscript, synthesizing data, executing a task, or brainstorming a grant application, the meeting must serve as a site of active synthesis rather than passive reporting. Teams do not arrive at synthesis by default (Halfon and Sovacool, 2023). Left unaddressed, these gaps can cause teams to remain at the level of information exchange, with members passively reporting on their own disciplinary progress without ever integrating that knowledge into a shared product. We define passive reporting as a meeting structure in which members take turns updating the group on their individual progress without engaging in explicit dialogue about how that progress connects to, challenges, or reshapes other members’ work. Active facilitation, by contrast, refers to structured interventions, such as differentiation and integration prompts which we discuss below, that require members to explicitly reconcile, connect, or challenge each other’s contributions in real time. This synthesis work is a hallmark of the team’s action phase, when members are no longer simply planning how to collaborate but are actively converting their distributed expertise into a shared product. To achieve this, we recommend employing active facilitation techniques specifically designed to bridge disciplinary boundaries. This can be operationalized by explicitly assigning the role of an Integrator, perhaps even rotating this role among members, whose sole responsibility is to operationalize the function of the meeting as an opportunity for Team Coaching (Shuffler et al., 2018). Hackman and Wageman (2005) defined team coaching as the “direct interaction with a team intended to help members make coordinated and task-appropriate use of their collective resources in accomplishing the team’s work” (p. 269). This approach moves the team beyond simple information sharing to actively weaving the team’s distributed knowledge into a cohesive whole by asking questions that will force explicit conversations regarding differentiation and knowledge integration.

This role does not necessarily require formal facilitation certification or specialized team coaching training, but it does require interpersonal expertise (Cravens et al., 2022); rather, what is critical is that the Integrator consistently asks the differentiation and integration prompts described below, prompts that can be structured in advance and don’t require advanced facilitation expertise. For example, during early ideation phases, the facilitator could employ differentiation prompts like asking team members, particularly those representing different disciplines or unique expertise, questions that surface hidden disciplinary assumptions. These differentiation prompts serve a function similar to the reflective, norm clarifying work of the transition phase, even when they occur within an ongoing action phase meeting. We know from the literature (Hall et al., 2012), that transdisciplinary teams must explicitly surface deep-seated disciplinary assumptions to prevent them from creating invisible barriers to mutual understanding that stall the development of a shared mental model. During synthesis phases, the facilitator must pivot to integration prompts, asking explicit questions about making connections among the knowledge shared to weave distributed insights into a cohesive whole, which represents the core work of the action phase for teams. Furthermore, effective facilitation requires managing the affective atmosphere and interpersonal nature of the meeting to ensure it remains conducive to creating connections. By attending to the emotional tone of the interaction, the facilitator can ensure that the vulnerability required for learning and cross-disciplinary exploration is supported rather than constrained.

**RECOMMENDATION 3**: Assign a rotating integrator role to move beyond passive reporting and to actively facilitating cross-disciplinary synthesis, thus creating meetings that are Team Coaching interventions.

### Maintaining the shared mental model of the group as an entity

The decay of shared understanding, where the team’s collective cognition and capabilities erode over time, begins the moment the meeting ends. As noted in the literature, decay is not merely the loss of individual knowledge; it is the erosion of team skills, the collective ability to maintain points of coordination, maintain collective identity and motivation, and execute processes effectively (DiazGranados et al., 2013). Further, scientific team members may have additional projects with which they participate. Therefore, part of the deterioration that occurs is their shared understanding of the group’s structure and even existence.

To combat this, we recommend moving beyond static meeting minutes to building a shared digital knowledge hub. Rather than a passive record, this should be a living, cloud-based workspace accessible to all members that captures not just what was decided, but why, specifically documenting how different disciplinary perspectives were synthesized to reach a conclusion. This hub establishes the clear roles and expectations necessary for preventing decay and it acts as the reinforcement mechanism that also counteracts decay, ensuring that the integrated knowledge generated during action-phase meetings survives the return to individual work and remains available as the team re-enters its next transition or action phase. By making this integration process visible and retrievable, the team creates a tangible, accessible record of their collective intelligence, further reinforcing their entitativity and shared purpose.

**RECOMMENDATION 4**: Counteract team decay between action and transition phases by establishing a shared knowledge hub to sustain shared mental models and reinforce the team’s entitativity between meetings.

## Future research directions and limitations

While we provide a conceptual framework (see Figure 1) for re-imagining meetings as embedded TDIs, empirical work is needed to validate these mechanisms, particularly as the nature of scientific work continues to evolve. We highlight three specific avenues for future inquiry that promise to advance the science of meetings and teams. First, as scientific collaboration becomes increasingly global and distributed, the coordination challenges we describe become even more acute in the virtual meeting space where the reliance on traditional video conferencing platforms (e.g., Zoom, Teams) often strip away the spatial and informal cues necessary for feeling like a cohesive entity rather than a collection of tiles on a screen. Moreover, the structuration process in virtual teams is even more difficult because shared environmental cues related to the team disappear when the meeting ends. Future research must examine how emerging immersive collaboration tools, such as Gather or virtual reality (VR) environments, affect the emergence of entitativity in fully distributed teams.

**FIGURE 1:**
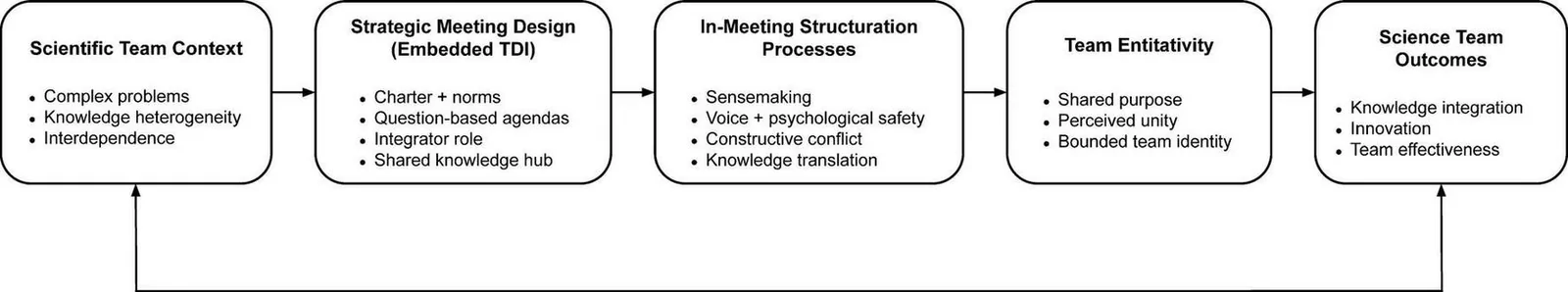
A conceptual framework for meetings as embedded Team Development Interventions.

Unlike standard video conferencing, these platforms reintroduce spatial agency, allowing members to move an avatar through a digital space, engage in serendipitous hallway conversations, and fluidly move between small-group ideation and large-group meetings. In addition, team members are more likely to perceive a collective shared space as their own, providing a digital anchor for their identity. We believe that contributing these spatial affordances may accelerate the development of entitativity by mimicking the physical proximity cues identified by Blanchard et al. (2020). Furthermore, the role of immersive VR warrants investigation. Does the sense of embodiment and presence provided by VR meetings create a stronger affective bond and shared identity than 2D video? Comparative, longitudinal studies that track entitativity and coordination outcomes across teams who utilize different platforms would help clarify which technological affordances most effectively offset the coordination costs of distributed collaboration. Research should explicitly test whether these high-immersion meeting modalities function as stronger structural interventions for reducing the decay of social cohesion in long-duration, distributed science collaborations.

Second, we call for a move beyond the common global meeting outcome measures such as meeting satisfaction or perceived effectiveness. To fully understand the meeting as a TDI, future research must rigorously link specific meeting design aspects to the affective, behavioral, and cognitive drivers of team performance (Salas et al., 2008). We need to understand the mechanisms through which meeting interactions build the team’s affective connections. For example, How do specific facilitation behaviors (e.g., active listening, checking for consensus) mechanistically build psychological safety and trust over time in science teams? Longitudinal studies could track how well-facilitated meetings buffer teams against the destructive relational conflict intrinsic in interdisciplinary disputes. Research should examine how specific artifacts, such as the question-based agenda or the shared digital knowledge hub directly influence the accuracy of shared mental models and transactive memory systems. Does the act of co-creating a meeting agenda actually improve the team’s shared understanding of who knows what? Finally, How do meeting norms translate into behavioral changes outside the meeting? Research is needed to link in-meeting behaviors (e.g., constructive debate) to post-meeting coordination (e.g., back-up behavior, error monitoring) within the team as well as to subsequent collaborations in other contexts. By deconstructing the meeting into these specific mechanistic pathways, scholars can provide evidence-based guidance that links meeting design to team specific outcomes.

Finally, the rapid entrance of artificial intelligence into meetings, including notetaker bots, real-time transcription systems, and increasingly human-like conversational avatars, signals a significant shift in how scientific teams coordinate and collaborate. On the positive side, these tools promise to streamline some cognitive burdens in meetings by automating routine tasks such as capturing minutes, identifying action items, and generating coherent summaries that support continuity across sessions. Yet these same capabilities introduce new complexities for research groups. When teams rely too heavily on AI to track discussions or represent absent members, participants may become more passive, inadvertently weakening the shared construction of meaning that underpins entitativity and collective understanding. The presence of AI avatars may shift expectations of what it means to “show up,” complicating norms of participation and psychological safety (Beck et al., 2025). Additionally, disparities in access to or comfort with AI tools across labs or institutions may create inequities in how teams coordinate, potentially amplifying existing divides in collaboration effectiveness. To tackle these issues, researchers might examine how AI-mediated meeting practices influence core meeting processes, from participation and decision-making to psychological distance and shared cognition. By studying these sociotechnical interactions systematically, meeting science can help ensure that AI becomes a complement, rather than a complication, to the collaborative work of scientific teams.

Several boundary conditions of the proposed framework also warrant explicit acknowledgment. First, our arguments are grounded in the context of scientific teams, whose work is characterized by high interdependence, specialized and distributed expertise, and may have lengthy project lifecycles. It remains an open question whether these recommendations generalize to non-scientific teams, whose collaborative demands and incentive structures may differ substantially. Second, while we discuss the possible power differences that may exist in science teams, our recommendations assume the ability to co-create in spite of power differences. In highly hierarchical teams, where status differences are pronounced or formally institutionalized, practices such as rotating the Integrator role or explicitly inviting sole discipline perspectives may be more difficult to implement without additional scaffolding. Third, several of our recommendations, including dedicated charter-building meetings, structured facilitation, and the maintenance of a shared digital knowledge hub, require a nontrivial investment of time, facilitation skill, and resources; teams operating under significant resource constraints, such as short-term grants, understaffed projects, or trainees without formal facilitation experience, may find these practices difficult to sustain.

## Conclusion

Scientific collaboration has been described as facing a coordination crisis, a struggle to integrate diverse, highly specialized knowledge across distributed networks to solve society’s most intractable problems (National Academies of Sciences, Engineering, and Medicine [NASEM], 2025). This crisis concerns not only the structural barriers to collaboration but also a fundamental devaluation of the primary mechanism through which science is actually conducted, the meetings science teams hold. In this paper, we have argued that the prevailing view of meetings as administrative drains on productivity is a dangerous fallacy that blinds us to their true function. We have re-conceptualized meetings not as logistical necessities, but as the essential structuring episodes where team entitativity is constructed and as the embedded interventions (TDIs) necessary to prevent the decay of productive knowledge integration and team shared mental models. The solution to the challenges of modern science is not to escape the meeting, but to reclaim it. By shifting our focus from the logistics of scheduling to the psychology of interaction, transforming meetings from passive reporting sessions into dynamic sites of knowledge integration, we offer a viable avenue for scientific teams to harness their full integrative capacity. Ultimately, the quality of the science cannot be separated from the quality of the interaction; to advance the former, we must rigorously redesign the latter.
